# Analysis of Sildenafil in Liquor and Health Wine Using Surface Enhanced Raman Spectroscopy

**DOI:** 10.3390/ijms20112722

**Published:** 2019-06-03

**Authors:** Shupei Xiao, Yong He

**Affiliations:** 1 College of Biosystems Engineering and Food Science, Zhejiang University, Hangzhou 310058, China; 180312@zju.edu.cn; 2 Key Laboratory of Spectroscopy Sensing, Ministry of Agriculture, Zhejiang University, Hangzhou 310058, China

**Keywords:** sildenafil, surface-enhanced Raman spectroscopy, health wine, liquor, limit of detection, quantitative determination, linear relationship

## Abstract

The illegal adulteration of sildenafil in herbal food supplements and alcoholic drinks immensely threatens human health due to its harmful side-effects. Therefore, it is important to accurately detect and identify the presence of sildenafil in alcoholic drinks. In this study, Opto Trace Raman 202 (OTR 202) was used as surface enhanced Raman spectroscopy (SERS) active colloids to detect sildenafil. The results demonstrated that the Raman enhancement factor (EF) of OTR 202 colloids reached 1.84 × 10^7^ and the limits of detection (LODs) of sildenafil in health wine and liquor were found to be as low as 0.1 mg/L. Moreover, the SERS peaks of 645, 814, 1235, 1401, 1530 and 1584 cm^−1^ could be qualitatively determined as sildenafil characteristic peaks and the relationship between Raman peak intensity and sildenafil concentration in health wine and liquor were different. There was a good linear correlation between Raman peak intensity, and sildenafil concentration in health wine ranged 0.1–1 mg/L (0.9687< *R*^2^ < 0.9891) and 1–10 mg/L (0.9701 < *R*^2^ < 0.9840), and in liquor ranged 0.1–1 mg/L (0.9662 < *R*^2^ < 0.9944) and 1–20 mg/L (0.9625 < *R*^2^ < 0.9922). The relative standard deviations (RSD) were less than 5.90% (sildenafil in health wine) and 9.16% (sildenafil in liquor). The recovery ranged 88.92–104.42% (sildenafil in health wine) and 90.09–104.55% (sildenafil in liquor). In general, the sildenafil in health wine and liquor could be rapidly and quantitatively determined using SERS technique, which offered a simple and accurate alternative for the determination of sildenafil in alcoholic drinks.

## 1. Introduction

Sildenafil is one of the inhibitors of phosphodiesterase type 5 (PDE-5), which is frequently added into health supplementary products for the treatment of erectile dysfunction (ED) in males [1]. Due to its side-effects such as headache, dyspepsia, back pain, rhinitis and flu syndrome, the usage of sildenafil must be strictly controlled by medical supervision [2,3]. Several investigations have indicated that sildenafil has been illegally adulterated into some dietary supplements, herbal medicines and alcoholic drinks [4,5,6,7]. Limited by the complicated sample processing and extraction, traditional methods for determining sildenafil mainly focus on high-performance liquid chromatography (HPLC) [8,9], ultraviolet spectrophotometry (UV) [10,11], gas chromatography-mass spectrometry (GC-MS) [12,13], thin layer chromatography (TLC) [14,15] and near infrared spectrometry (NIR) [16,17]. Ramirez et al. [18] quantified plasma levels of sildenafil and its metabolite by liquid chromatography (LC) with a C_18_ reverse-phase column and UV detection; the limits of detection (LODs) and quantification were 1 and 10 ng/mL, respectively. Oris et al. [19] determined ardenafil, sildenafil, tadalafil, testosterone, procaine, lidocaine, prilocaine, and benzocaine in cosmetic creams by using HPLC method with ultraviolet diode array (UV-DAD) and electrospray ionization mass spectrometry (ESI-MS). The results show a good determination coefficient (*R*^2^ ≥ 0.99) and the limits of quantification range 2.5–7.8 μg/g and 3.3–8.9 ng/g for HPLC-UV-DAD assay and HPLC-ESI-MS assay, respectively. Kee et al. [20] differentiated two groups of PDE-5 inhibitors including four sildenafil- and three thiosildenafil-like analogs by Orbitrap-mass spectrometry, indicating that the identification of PDE-5 analogs in dietary supplements can be possibly done in a wide concentration range. Liew et al. [21] applied ultra-fast liquid chromatography (UFLC) with ESI-MS/MS to quantify sildenafil and *N*-desmethyl sildenafil. The linear concentration ranges of sildenafil and *N*-desmethyl sildenafil are 10–800 ng/mL and 10–600 ng/mL, respectively, and the correlation coefficients are *R*^2^ ≥ 0.9976 and *R*^2^ ≥ 0.9992 respectively. Yaroshenko et al. [22] determined the sildenafil in blood plasma using HPLC with UV and mass-spectrometry (MS). The results show that the limits of quantification of sildenafil are 20 and 5 ng/mL using HPLC-UV and HPLC-MS methods, respectively.

Although the traditional methods for determining sildenafil achieve high sensibility, the complex and time-consuming sample pretreatment process, huge instruments and high cost limit their developments. Compared with the methods mentioned above, surface enhanced Raman spectroscopy (SERS) shows great potential on ultrasensitive and label-free chemical or biochemical analysis based on its advantages of simple sample treatment and fast sample screening speed [23,24]. Recently, some researchers applied SERS technique for the determination of sildenafil and its analogs. Lv et al. [25] proposed a SERS method based on a solid-phase dendritic Ag nanostructure for the trace detection of sildenafil, and the superiority in practical application of SERS technique is verified through the Raman spectroscopy (RS) of sildenafil within 1150–1699 cm^−1^. Liu et al. [15] investigated the characteristics and influencing factors of sildenafil on the thin layers chromatographic surface-enhanced Raman spectroscopy (TLC-SERS). The results suggest that the peaks at 1563, 1530, 1405, 1240 and 1272 cm^−1^ can be determined as sildenafil characteristic peaks. They did not mention the LOD of sildenafil. Mao et al. [26] used the micro-Raman spectroscopy as a non-destructive technique to screen sildenafil and tadalafil adulterated in the healthcare products, and confirms the detection results with wavelet transform (WT) by LC/MS measurements. Zhao et al. [27] used SERS technique to detect the illegally-added sildenafil in drugs. The results show that the sildenafil in aqueous solutions as low as 1 mg/L can be semi-quantitatively detected with high signal uniformity (RSD = 3.77%). Zhang et al. [28] applied SERS analysis method for the simultaneous detection of five types of illegal chemical drugs added in Chinese proprietary medicines. Wu et al. [29] applied SERS with sodium alginate (SA)-silver nanoparticles (AgNPs) substrates for the determination of vardenafil and rosiglitazone maleate (ROS) in natural healthcare products. The LOD and ROS of vardenafil are as low as 1.63 and 2.20 mg/L, respectively. Lin et al. [30] applied SERS to detect sildenafil in cocktail, and the LOD reaches 0.1 mg/L. However, in practice, there are few cases of sildenafil in cocktails, while there are many studies based on sildenafil determination in liquor and health wine. Although the partial least squares (PLS) nonlinear model effect is good, for the quantitative detection of SERS, the single peak used in this study was more convincing.

Based on the analysis mentioned above, it is important to accurately detect sildenafil in alcoholic drinks such as health wine and liquor using SERS. In this study, the Opto Trace Raman 202 (OTR 202) was used as SERS active colloids. The characterization of OTR 202 colloids were studied and the Raman enhancement factor (EF) of OTR 202 colloids was calculated. Besides, the relationship between Raman peak intensity and sildenafil concentration in health wine and liquor were established to realize the quantitative determination of sildenafil.

## 2. Results and Discussion

### 2.1. Characterization of Opto Trace Raman 202

**Figure 1:**
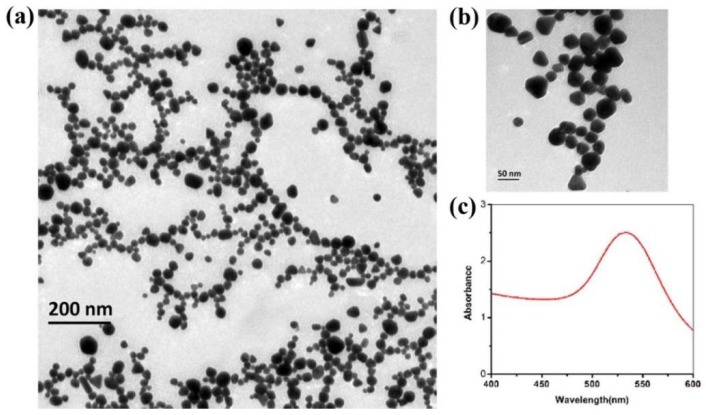
Transmission electron microscopy (TEM) images of Opto Trace Raman 202 (OTR 202) at: 200 nm scale (**a**); and 50 nm scale (**b**). The ultraviolet spectrophotometry (UV) spectroscopy of OTR 202 (**c**).

Figure 1a,b presents representative TEM images of Opto Trace Raman 202 colloids at 200 nm scale and 50 nm scale, respectively. The UV spectroscopy of OTR 202 colloids is given in Figure 1c.

It can be clearly seen that the diameter of OTR 202 colloids was not very uniform and the shape of OTR 202 colloids was not a regular sphere. The diameter of OTR 202 colloids was in the range of 20–50 nm and the UV/Visible characteristic absorption peaks of OTR 202 colloids was at 533 nm within the band of gold nanoparticles (AuNPs) ranging from 450 to 600 nm [31] (Figure 1c). Compared with the results of Dong et al. [32,33] (AuNPs: 27.8 nm; UV/Visible characteristic absorption peaks: 543 nm), Luo et al. [34] (AuNPs: 23–102 nm; UV/Visible characteristic absorption peaks: 525–549 nm) and He et al. [24] (AuNPs: 41–50 nm; UV/Visible characteristic absorption peaks: 525–540 nm), we could infer that the properties of OTR 202 colloids were consistent with AuNPs colloids. Therefore, the OTR 202 colloids was suitable as SERS substrate in this paper. Compared with Lin’s [30] study, the properties of OTR 202 data were more convincing.

### 2.2. The SERS Performance of Rhodamine 6G Substrate

**Figure 2:**
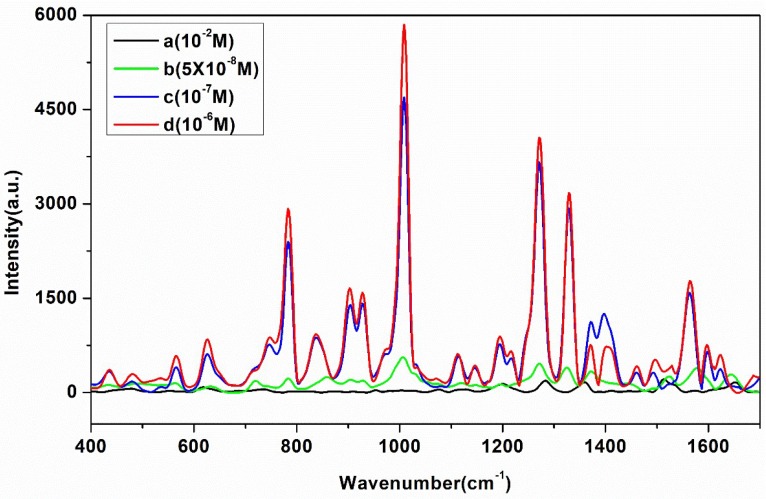
(a) Raman spectroscopy (RS) of 10^−2^ M Rhodamine 6G (R6G); and surface enhanced Raman spectroscopy (SERS) spectra of R6G with different concentrations: (b) 5 × 10^−8^ M; (c) 10^−7^ M; and (d) 10^−6^ M.

In this study, Rhodamine 6G (R6G) was used as a probe to investigate SERS activity of OTR 202 colloids. Furthermore, the SERS spectra of R6G with different concentrations were obtained, as shown in Figure 2.

As shown in Figure 2, the RS of 10^−2^ M R6G only had a faint signal. However, the LOD of R6G could reach 5 × 10^−8^ M when the R6G was mixed with OTR 202 colloids. The Raman EF was used to measure the enhancement effect, which was calculated as follows [35]:

$$\mathrm{EF}=\frac{I_{\mathrm{SERS}}C_{\mathrm{Raman}}}{I_{\mathrm{Raman}}C_{\mathrm{SERS}}}$$

where $I_{\mathrm{SERS}}$ is the integrated intensity of R6G molecules adsorbed on the colloids surface; $I_{\mathrm{Raman}}$ is the integrated intensity of the same Raman band obtained without the OTR 202 colloids; $C_{\mathrm{SERS}}$ represents the concentration of R6G adsorbed on OTR 202 colloids; and $C_{\mathrm{Raman}}$ represents the concentration of R6G that can be detected by ordinary RS.

In this work, the values of $C_{\mathrm{SERS}}$ and $C_{\mathrm{Raman}}$ were 10^−7^ M and 10^−2^ M, respectively. The conditions for Raman and SERS measurement were kept constant. The ratio of $I_{\mathrm{SERS}}$/$I_{\mathrm{Raman}}$ from the Raman intensities at 784, 1010, 1272 and 1330 cm^−1^ were calculated, as shown in Table 1. It can be clearly seen that the OTR 202 colloids exhibited high EF of R6G, especially at 1331 cm^−1^ with an EF of 1.84 × 10^7^, which indicated that the SERS had high sensitivity and stability based on OTR 202 colloids.

### 2.3. The SERS Performance of Sildenafil

**Figure 3:**
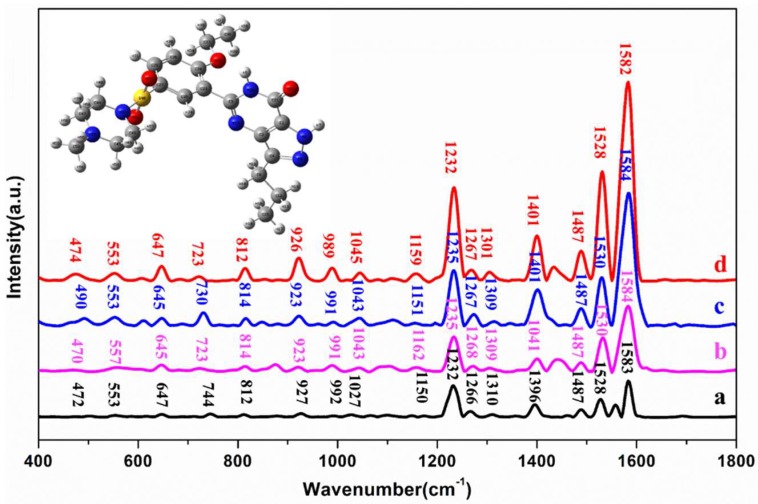
(a) RS of powder sildenafil; (b) SERS spectra of sildenafil in liquor; (c) SERS spectra of sildenafil in health wine; and (d) SERS spectra of sildenafil solution mixed with methanol. Inset: The molecular structure of sildenafil.

To investigate the sensitivity and stability of the OTR 202 colloids for the detection of sildenafil in health wine and liquor, the normal RS of sildenafil (molecular formula: C_22_H_30_N_6_O_4_S) powder, the SERS of 100 mg/L solution mixed with methanol, the SERS of 100 mg/L sildenafil in liquor, and the SERS of 100 mg/L sildenafil in health wine were collected, as shown in Figure 3. The detailed vibrational modes of sildenafil are listed in Table 2.

Clearly, the SERS signals of sildenafil solution were stronger than those of sildenafil in liquor and health wine. The normal Raman peaks of sildenafil were in good agreement with the previous literature [27], and the SERS of sildenafil in liquor and health wine were basically similar to the SERS of sildenafil solution, whose Raman shifts (less than 10 cm^−1^) were within a reasonable range, which indicated that the position of SERS peaks detected by SERS spectra based on OTR 202 colloids were feasible and reliable.

According to density functional theory (DFT) calculation based on the B3LYP/6-31G (d,p) method in Gaussian v.09 software [36], the peaks at 472 and 553 cm^−1^ could be assigned to carbonyl stretching and phenetole breathing deformable vibration; the peaks at 647 and 723 cm^−1^ were derived from the carbonyl stretching, phenetole deformable vibration and C–S stretching in sulfamide; 812 cm^−^^1^ belonged to the pyrazole pyridine stretching; the peaks located at 926 and 1027 cm^−1^ were all related to C–C deformable vibration and C–H stretching in pyrazole pyridine group; the peaks at 989, 1159 and 1232 cm^−^^1^ were the C–H stretching vibration in carbonyl; 1310 cm^−1^ was C–H stretching vibration in ethyl; 1401 cm^−^^1^ was the C–H deformable vibration in methyl piperazine; and 1487, 1528 and 1582 cm^−^^1^ were the C–H deformable vibration in pyrazole pyridine. 

### 2.4. Detection of Sildenafil in Health Wine and Liquor

**Figure 4:**
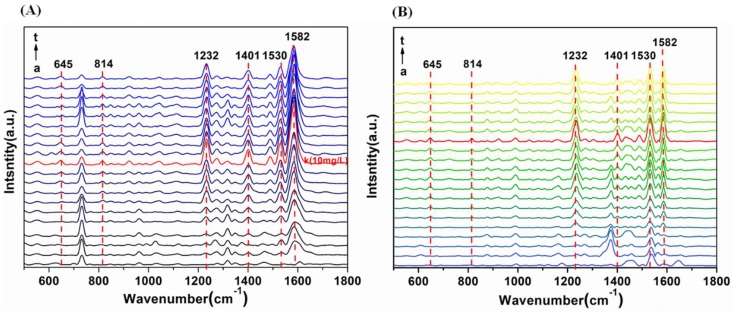
SERS spectra of sildenafil in health wine (**A**) and liquor (**B**) with different concentrations (from a to t: 0, 0.1, 0.2, 0.4, 0.6, 0.8, 1, 2, 4, 6, 8, 10, 15, 20, 25, 30, 35, 40, 45 and 50 mg/L, respectively).

**Figure 5:**
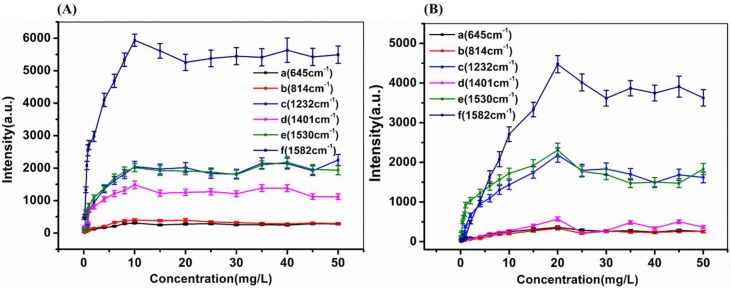
The SERS peak intensities of sildenafil concentration ranging from 0.1 mg/L to 50 mg/L in health wine (**A**) and liquor (**B**) at: 645 cm^−1^ (a); 814 cm^−1^ (b); 1232 cm^−1^ (c); 1401 cm^−1^ (d); 1530 cm^−1^ (e); and 1582 cm^−1^ (f).

Furthermore, we investigated the accuracy and stability of OTR 202 colloids for the detection of sildenafil in health wine and liquor. The representative SERS spectra of sildenafil in health wine and liquor with different concentrations are given in Figure 4. The SERS of sildenafil concentration ranged from 0 to 50 mg/L (0, 0.1, 0.2, 0.4, 0.6, 0.8, 1, 2, 4, 6, 8, 10, 15, 20, 25, 30, 35, 40, 45 and 50 mg/L) in health wine and liquor were obtained and the SERS peaks at 645, 814, 1232, 1401, 1530 and 1582 cm^−1^ are shown in Figure 5.

As shown in Figure 4 and Figure 5, with the increase of sildenafil in health wine from 0.1 mg/L to 50 mg/L, the SERS peaks at 645, 814, 1232, 1401, 1530 and 1582 cm^−1^ increased sharply within the range of 0.1–10 mg/L, while they slowly increased when the sildenafil concentration continued to increase from 10 to 50 mg/L. In addition, when the sildenafil concentration in health wine increased from 10 to 50 mg/L, the intensities of SERS peaks at 645, 814, 1232, 1401, 1530 and 1582 cm^−1^ basically did not change. It was found that the sildenafil in health wine could still be identified even when the sildenafil solution concentration was as low as 0.1 mg/L. Compared with the previous research [27,29] with LODs of 1, 1.63 and 2.20 mg/L, the LOD of sildenafil in this study was improved greatly; both were far lower than 1%. Thus, the presented method can be successfully applied for the quantification of real natural healthcare products. In addition, there were SERS peaks at 730, 940 and 1310 cm^−1^ when the sildenafil was not added in health wine (Figure 5Aa). Although these peaks belonged to sildenafil as well, they might be the Raman characteristic peaks of some kind of traditional Chinese medicines in health wine. 

**Figure 6:**
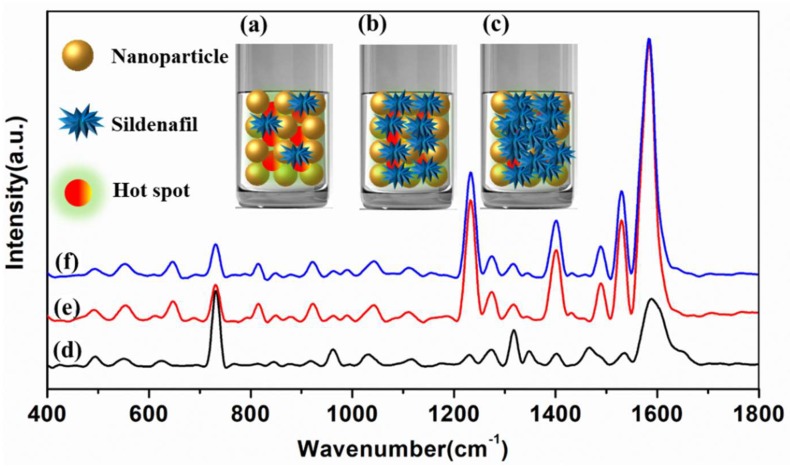
The diagram of the “hot spots” among the sildenafil molecules and nanoparticles. The diagram of the “hot spots” among the nanoparticles with low (**a**), appropriate (**b**) and extraordinary high (**c**) sildenafil concentration. The SERS spectra with low (**d**), appropriate (**e**) and extraordinary high (**f**) sildenafil concentration.

Similar to the SERS spectra of sildenafil in health wine, the SERS peaks at 645, 814, 1232, 1401, 1530 and 1582 cm^−1^ increased rapidly when the sildenafil concentration in liquor was increased from 0.1 to 20 mg/L, while those SERS peaks fluctuated greatly in the range of 20–50 mg/L. Moreover, it can be clearly seen that the SERS peak intensities of sildenafil in health wine were generally higher than that in liquor, which indicated that the background difference between health wine and liquor would lead to the difference in SERS characteristic peak intensities. In conclusion, 645, 814, 1232, 1401, 1530 and 1582 cm^−1^ could be qualitatively determined as sildenafil characteristic peaks in health wine and liquor, and a possible reason is shown in Figure 6.

It is well known that the SERS signals can be greatly enhanced by the so-called “hot spots” effect. According to Figure 6, with the constant amount of “hot spots”, the “hot spot” effect among the nanoparticles were not completed when the sildenafil concentration was low (Figure 6a). The “hot spot” effect among the nanoparticles gradually increased with the increase of sildenafil concentration, resulting in the rapid enhancement of SERS signals (Figure 6b). When the concentration of sildenafil increased to a certain extent, the “hot spot” effect among the nanoparticle particles tended to be saturated, thus the enhancement effect of the SERS signal was not obvious (Figure 6c).

### 2.5. Quantitative Detection of Sildenafil in Health Wine and Liquor

**Figure 7:**
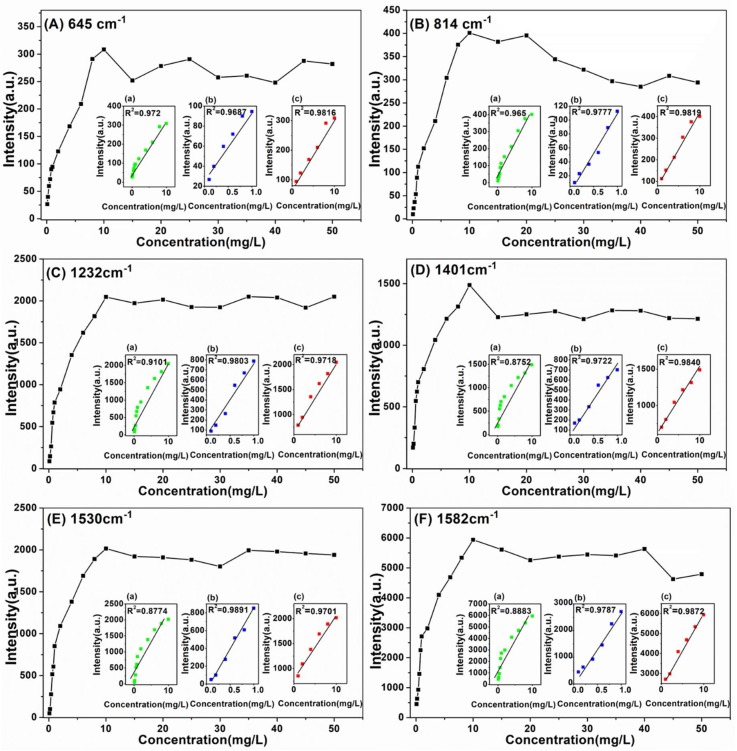
The intensities of SERS peak versus sildenafil concentration in health wine at: 645 cm^−1^ (**A**); 814 cm^−1^ (**B**); 1232 cm^−1^ (**C**); 1401 cm^−1^ (**D**); 1530 cm^−1^ (**E**); and 1582 cm^−1^ (**F**). Inset: The linear calibration plotted in the concentration range: (a) 0.1–10 mg/L; (b) 0.1–1 mg/L; and (c) 1–10 mg/L.

**Figure 8:**
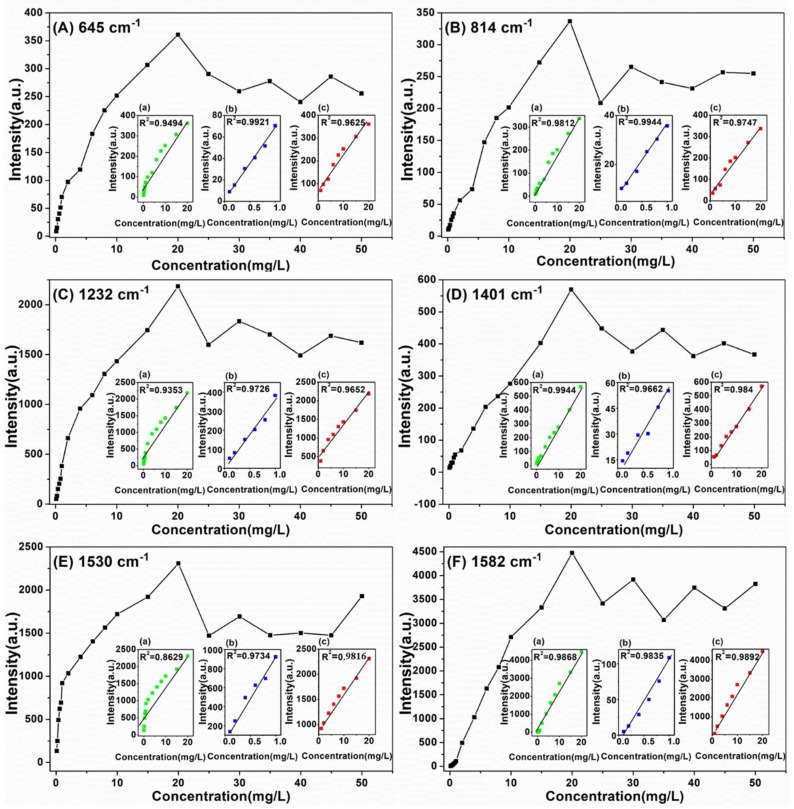
The intensities of SERS peaks versus sildenafil concentration in liquor at: 645 cm^−1^ (**A**); 814 cm^−1^ (**B**); 1232 cm^−1^ (**C**); 1401 cm^−1^ (**D**); 1530 cm^−1^ (**E**); and 1582 cm^−1^ (**F**). Inset: The linear calibration plotted in the concentration range: (a) 0.1–20 mg/L; (b) 0.1–1 mg/L; and (c) 1–20 mg/L.

Moreover, the relationship between Raman peak intensity at 645, 814, 1232, 1401, 1530 and 1582 cm^−1^ and sildenafil concentration ranging from 0.1 to 50 mg/L (0.1, 0.2, 0.4, 0.6, 0.8, 1, 2, 4, 6, 8, 10, 15, 20, 25, 30, 35, 40, 45 and 50 mg/L) in health wine (Figure 7) and liquor (Figure 8) were established. In addition, the linear equations at 645, 814, 1232, 1401, 1530 and 1582 cm^−1^ are given in Table 3 and Table 4, respectively.

According to Figure 7 and Table 3, there was a good linear correlation between Raman peak intensity and sildenafil concentration in health wine in each linear regression equation ranging 0.1–1 mg/L (0.9687 < *R*^2^ < 0.9891) and 1–10 mg/L (0.9701 < *R*^2^ < 0.9840). Although there was a good linear correlation in the range of 0.1–10 mg/L at 645 and 814 cm^−1^, the correlation coefficients were below 0.92 at 1232, 1401, 1530 and 1582 cm^−1^. Therefore, the sildenafil in health wine could be accurately and quantitatively detected by adopting different model in the range of 0.1–10 mg/L.

According to Figure 8 and Table 4, there was a good linear correlation between Raman peak intensity and sildenafil concentration in liquor in each linear regression equation ranging 0.1–1 mg/L (0.9662 < *R*^2^ < 0.9944) and 1–20 mg/L (0.9625 < *R*^2^ < 0.9922). Although there was a good linear correlation at 814, 1401 and 1582 cm^−1^, the correlation coefficients were only 0.9494, 0.9353 and 0.8629 at 645, 1232 and 1530 cm^−1^, respectively. Therefore, the sildenafil in liquor could be accurately and quantitatively detected by adopting different models in the range of 0.1–20 mg/L.

From the above analysis, although there were some differences in the detection of sildenafil in health wine and liquor by SERS technique, there was a good linear relationship between the intensity and concentration of different Raman characteristic peaks in a certain concentration range, which indicated that it was feasible to use SERS technology for the detection of sildenafil in both health wine and liquor.

### 2.6. Model Accuracy Verification

To verify the accuracy of this detection method of sildenafil, first different concentrations of sildenafil in health wine (0.5, 5 mg/L) and liquor (0.5, 5 and 13 mg/L) were prepared; each concentration contained nine samples. Second, all samples were detected by SERS based on OTR 202 colloids. Third, the linear regression equations at 645, 814, 1232, 1401, 1530 and 1582 cm^−1^ were used to predict the sildenafil concentration in health wine and liquor. Table 5 and Table 6 present the precision and accuracy for the determination of sildenafil in health wine and liquor, respectively.

According to Table 5, the sildenafil concentration in health wine could be well predicted using the linear regression equations at 645, 814, 1232, 1401, 1530 and 1582 cm^−1^. The relative standard deviation (RSD) was less than 6.78% and 7.65% for two added concentrations, respectively. The recoveries were in the ranges of 88.92–104.42% and 97.62–100.43%, respectively. Moreover, the linear regression equations at 1530 cm^−1^ had the best precision and accuracy for 0.5 mg/L (predicted + SD: 0.480 ± 0.013; RSD: 2.89; Recovery: 96.11), 5 mg/L (predicted + SD: 5.02 ± 0.13; RSD: 2.53; Recovery: 100.43) and 13 mg/L.

According to Table 6, the sildenafil concentration in liquor could also be well predicted using the linear regression equations at 645, 814, 1232, 1401, 1530 and 1582 cm^−1^. The RSD were less than 8.07%, 8.70% and 7.68%, respectively, at the three added concentrations. The recoveries were in the ranges of 92.77–104.45%, 90.09–102.80% and 92.99–101.84%, respectively. Moreover, the linear regression equations at 1232 cm^−1^ had the best precision and accuracy for 0.5 mg/L (predicted + SD: 0.498 ± 0.022; RSD: 4.59; Recovery: 99.78), 5 mg/L (predicted + SD: 4.93 ± 0.23; RSD: 4.88; Recovery: 98.60) and 13 mg/L (predicted + SD: 13.24 ± 0.63; RSD: 4.66; Recovery: 101.84). From the results above, we conclude that the application of our method for sildenafil detection has excellent practical value.

## 3. Materials and Methods

### 3.1. Chemicals

The reagents used in this experiment included sildenafil (99.8% purity, Sigma-Aldrich, Beijing, China), methanol (chromatographically pure, Amethyst Chemicals, Beijing, China), R6G (96.01% purity, Sigma-Aldrich, Beijing, China), health wine (Jinpai Co., Ltd., DaYe, China) and liquor (Beijing Red Star Co., Ltd. Beijing, China). In addition, the OTR 202 colloids produced by Opto Trace Technologies, Inc. (Suzhou, China) were used as SERS substrate in this study.

### 3.2. Instruments

The experimental instruments included: (1) RmTracer-200-HS portable Raman spectrometer combined with a 785 nm excitation wavelength diode-stabilized stimulator (Opto Trace Technologies, Inc., Mountain View, CA, USA); (2) FEI Tecnai G2 F20 S-TWIN transmission electron microscope (FEI Company, Hillsboro, OR, USA); and (3) Vortex-Genie 2/2T vortex mixer (Shanghai Ling early Environmental Protection Instrument Co., Ltd, Shanghai, China).

### 3.3. Sample Preparation

The sample preparation process was as follows. First, the standard sildenafil was diluted to 1000 mg/L with methanol. Second, the standard solution of 1000 mg/L was diluted to 0–50 mg/L with health wine and liquor, respectively. For each concentration the sample was prepared in triplicate.

### 3.4. SERS Measurement

Before Raman spectra acquisition, the instrument was calibrated using a 785 nm excitation wavelength. The parameters were set as follows: a power of 200 mw, a scanning range of 200–3300 cm^−1^, an optical resolution of 2 cm^−1^, an integration time of 10 s and an average spectral value of 3 times. When collecting the SERS of samples, 500 μL OTR 202 colloids and 100 μL test solution were added in turn into a 2 mL quartz bottle, and then it was placed at a liquid sample pool.

## 4. Conclusions

In this paper, we report a rapid and quantitative determination method of sildenafil in health wine and liquor based on SERS with OTR 202 colloids. We found that the Raman EF of OTR 202 colloids could reach 1.84 × 10^7^ and the proposed method showed good performance for sildenafil in health wine and liquor detection and the LODs were found to be as low as 0.1 mg/L; both were far lower than 1%. Therefore, the presented method can be successfully applied for the quantification of natural healthcare products. Moreover, there was a good linear correlation between Raman peak intensity and sildenafil concentration in both health wine (*R*^2^ = 0.9891) and liquor (*R*^2^ = 0.9944) at certain concentration ranges. It was indicated that the application of SERS technique for the rapid detection of sildenafil was feasible and reliable. Overall, the SERS method with OTR 202 colloids enhancement developed through this study provides a novel, rapid and accurate approach to quantitatively determine sildenafil in health wine and liquor, which could meet the requirements of sildenafil determination in other alcoholic drinks.

## Figures and Tables

**Table 1:** The enhancement factor (EF) of R6G based on OTR 202 colloids.

Wavenumber (cm^−1^)	$I_{Raman}$	$I_{SERS}$	$I_{SERS}$ $/I_{Raman}$	$C_{SERS}$ $/C_{Raman}$	EF
785	17	2377	140	10^5^	1.40 × 10^7^
1009	32	4697	149		1.49 × 10^7^
1271	102	3665	36		3.6 × 10^6^
1331	16	2928	184		1.84 × 10^7^

**Table 2:** The proposed assignment of sildenafil Raman peaks.

RS of Sildenafil Powder (cm^−1^)	SERS of Sildenafil Solution (cm^−1^)	SERS of Sildenafil in Liquor (cm^−1^)	SERS of Sildenafil in Health Wine (cm^−1^)	Assignments
472	474	470	490	υ carbonyl + δ phenetole
553	553	557	553	υ carbonyl + δ phenetole
647	647	645	645	υ carbonyl + δ phenetole + υ (C–S)
744	723	723	730	υ carbonyl + δ phenetole + υ (C–S)
812	812	814	814	υ Pyrazole pyridine
926	926	923	923	δ (C–C) + υ (C–H)
992	989	991	991	υ (C–H) in carbonyl
1027	1045	1043	1043	δ (C–C) + υ (C–H)
1150	1159	1162	1151	υ (C–H) in carbonyl
1232	1232	1232	1232	υ (C–H) in carbonyl
1310	1301	1309	1309	δ (C–H) in ethyl
1396	1401	1401	1401	δ (C–H) in methyl piperazine
1487	1487	1487	1487	δ (C–H) in Pyrazole pyridine
1528	1528	1530	1530	υ (C–H) in Pyrazole pyridine
1583	1582	1582	1582	δ (C–H) in Pyrazole pyridine

**Table 3:** The linear equation at 645, 814, 1232, 1401, 1530 and 1582 cm^−1^ of health wine.

Peaks (cm^−^^1^)	Linear Equation	Range (mg/L)	*R* ^2^	Peaks (cm^−1^)	Linear Equation	Range (mg/L)	*R* ^2^
645	*y* = 27.484*x* + 52.013	0.1–10	0.9720	814	*y* = 39.996*x* + 40.358	0.1–10	0.9650
	*y* = 76.502*x* + 24.209	0.1–1	0.9687		*y* = 112.35*x* − 4.0499	0.1–1	0.9777
	*y* = 24.832*x* + 70.683	1–10	0.9816		*y* = 33.846*x* + 84.522	1–10	0.9819
1232	*y* = 186.6*x* + 373.08	0.1–10	0.9101	1401	*y* = 120.74*x* + 403.59	0.1–10	0.8752
	*y* = 823.83*x* − 9.648	0.1–1	0.9803		*y* = 638.58*x* + 98.242	0.1–1	0.9722
	*y* = 140.39*x* + 702.58	1–10	0.9822		*y* = 86.188*x* + 649.19	1–10	0.9840
1530	*y* = 190.96*x* + 377.12	0.1–10	0.8801	1582	*y* = 520.22*x* + 1295.2	0.1–10	0.8883
	*y* = 891.26*x* − 60.683	0.1–1	0.9891		*y* = 2587.6*x* + 68.153	0.1–1	0.9787
	*y* = 129.74*x* + 816.6	1–10	0.9701		*y* = 365.32*x* + 2403.7	1–10	0.9872

**Table 4:** The linear equation at 645, 814, 1232, 1401, 1530 and 1582 cm^−1^ of liquor.

Peaks (cm^−1^)	Linear Equation	Range (mg/L)	*R* ^2^	Peaks (cm^−1^)	Linear Equation	Range (mg/L)	*R* ^2^
645	*y* = 29.237*x* + 6.7438	0.1–20	0.9921	814	*y* = 17.003*x* + 18.855	0.1–20	0.9812
	*y* = 65.767*x* + 2.1908	0.1–1	0.9944		*y* = 29.237*x* + 6.749	0.1–1	0.9944
	*y* = 15.437*x* + 74.515	1–20	0.9625		*y* = 16.09*x* + 30.708	1–20	0.9747
1232	*y* = 106.26*x* + 251.16	0.1–20	0.9353	1401	*y* = 27.004*x* + 20.516	0.1–20	0.9944
	*y* = 342.03*x* + 10.409	0.1–1	0.9726		*y* = 43.907*x* + 9.5066	0.1–1	0.9662
	*y* = 86.896*x* + 503.1	1–20	0.9652		*y* = 26.437*x* + 27.85	1–20	0.9922
1530	*y* = 96.516*x* + 593.72	0.1–20	0.8629	1582	*y* = 232.12*x* + 11.709	0.1–20	0.9896
	*y* = 824.13*x* + 93.493	0.1–1	0.9734		*y* = 112.76*x* − 11.609	0.1–1	0.9835
	*y* = 70.364*x* + 931.57	1–20	0.9816		*y* = 223.49*x* + 138.28	1–20	0.982

**Table 5:** The precision and accuracy for the determination of sildenafil in health wine.

Sildenafil Peaks in Health Wine (cm^−1^)		Predicted (mg/L) Mean + ^a^ SD					
		645	814	1232	1401	1530	1582
Added (mg/L)	0.5	0.472 ± 0.026	0.522 ± 0.021	0.467 ± 0.031	0.470 ± 0.024	0.480 ± 0.013	0.444 ± 0.029
^a^ RSD (%)		5.51	3.92	6.78	5.14	2.89	6.66
Recovery (%)		94.41	104.42	93.5	94.08	96.11	88.92
Added (mg/L)	5	4.89 ± 0.51	4.97 ± 0.37	4.93 ± 0.22	4.84 ± 0.22	5.02 ± 0.13	4.88 ± 0.42
^a^ RSD (%)		5.11	7.65	4.51	4.62	2.53	4.21
Recovery (%)		97.87	99.38	98.60	96.89	100.43	97.62

^a^ SD (standard deviation); RSD (relative standard deviation).

**Table 6:** The precision and accuracy for the determination of sildenafil in liquor.

Sildenafil Peaks in Health Liquor (cm^−1^)		Predicted (mg/L) Mean + SD					
		645	814	1232	1401	1530	1584
Added (mg/L)	0.5	0.483 ± 0.039	0.522 ± 0.036	0.498 ± 0.022	0.512 ± 0.043	0.534 ± 0.026	0.464 ± 0.025
^a^ RSD (%)		8.07	6.98	4.59	8.35	4.95	5.44
Recovery (%)		96.61	104.45	99.78	102.32	106.78	92.77
Added (mg/L)	5	4.71 ± 0.32	4.77 ± 0.28	4.93 ± 0.23	4.50 ± 0.34	5.14 ± 0.34	4.89 ± 0.43
^a^ RSD (%)		6.67	5.90	4.88	7.62	6.70	8.70
Recovery (%)		94.31	95.44	98.60	90.09	102.80	97.86
Added (mg/L)	13	12.78 ± 0.85	12.08 ± 0.89	13.24 ± 0.63	12.33 ± 0.90	12.70 ± 0.97	12.69 ± 0.98
^a^ RSD (%)		6.72	7.38	4.66	7.61	7.62	7.68
Recovery (%)		98.36	92.99	101.84	94.90	97.73	97.64

^a^ SD (standard deviation); RSD (relative standard deviation).
